# Implementing Digital Care Pathways and Changes in Contact Volume, Contact Distribution, and Outpatient Costs in Specialized Health Care: Retrospective Longitudinal Observational Study

**DOI:** 10.2196/97853

**Published:** 2026-10-27

**Authors:** Elina Laukka, Paulus Torkki, Taina Ala-Nikkola, Jussi Tikkanen, Anna Mason, Taru Lehtonen, Atte Meretoja

**Affiliations:** 1 The Wellbeing Services County of Ostrobothnia Vaasa Finland; 2 Department of Public Health Faculty of Medicine University of Helsinki Helsinki Finland; 3 Department of Industrial Engineering and Management LUT University Lappeenranta Finland; 4 HUS, Helsinki University Hospital and Helsinki University Helsinki Finland; 5 Faculty of Social Sciences, Health Sciences Tampere University Tampere Finland

**Keywords:** digitalization, digital care pathway, health care, visits, costs

## Abstract

**Background:**

Health care systems increasingly promote patient engagement and self-management through digital health services. Digital care pathways, which support coordinated care delivery across the patient journey, have emerged as a promising approach, although evidence regarding their impact on resource use and costs remains limited.

**Objective:**

The aim of this study was to examine the implementation and impact of digital care pathways specifically designed to reduce or replace physical contacts or phone consultations in specialized health care.

**Methods:**

We analyzed the 10 most widely used digital care pathways by user volume. The selection criteria were (1) more than 1500 users per digital care pathway and (2) the aim of replacing or reducing in-person contacts. Descriptive analyses quantified digital contact volumes, average contacts and costs per patient, and inflation-adjusted expenditures using the consumer price index, both overall and by patient group. Ecologic group-level correlations were examined to assess whether the 2024 proportion of digital contacts was associated with changes in physical contacts and total costs from 2018 to 2024.

**Results:**

Digital care pathways were progressively adopted between 2018 and 2024, increasing from no use to accounting for 19.1% (34,837/182,188) of all outpatient contacts among the studied patient groups. Physical visits declined from 74.8% (99,201/132,622) to 54.1% (98,642/182,188), while contacts per patient rose by 7.6% on average. Conditions with the highest uptake of digital pathways showed the strongest reductions in the share of physical visits per patient, with declines of up to 39.7% (0.73/0.44). Average outpatient costs per patient decreased by 18.2% (€421/€515; €1=US $1.12 as of Oct 16, 2026) in real terms, with a 39.3% (€233/€384) reduction in the costs of physical contacts and a 15.4% (€135/€117) increase in the costs of phone visits. Digital pathway contacts accounted for 12.6% (€53/€421 per patient) of total outpatient costs in 2024; however, only 8.8% of the variance in total cost levels was explained by digital pathway adoption, suggesting that the shift toward digital care pathways did not substantially drive overall cost changes.

**Conclusions:**

Digital care pathways expanded rapidly, reaching nearly one-fifth of all outpatient contacts by 2024 in the included patient groups and contributing to a notable reduction in physical visits. However, their overall cost impact remains uncertain, as there have also been other factors impacting the development of outpatient costs.

## Introduction

Health care systems increasingly strive to empower patients to take an active role in managing their own health and well-being [1]. Digital health services are widely promoted as a way to deliver high-quality care more cost-effectively and to improve access [2]. To meet diverse health and system needs, new digital health solutions are continuously developed, implemented, evaluated, and refined using information and communication technologies [3]. Supporting this broader shift, digital health services, and particularly digital care pathways, have emerged as promising tools for enhancing patient engagement and care delivery [4,5]. In this study, digital health services are understood as a broad category of health care services enabled or supported by digital technologies [6]. The present study focuses specifically on digital care pathways, a subtype of digital health service that supports and coordinates defined care processes through integrated digital functionalities. Unlike stand-alone digital tools or communication channels, digital care pathways are designed to support coordinated care delivery across the patient care journey through integrated digital functionalities [7]. In the Finnish health care context, a digital care pathway is defined as a secure digital service channel for patients who have an established care relationship with a health care provider [8]. They provide an effective and scalable approach for routinely gathering patient-reported outcomes, with the potential to support continuous remote care [9]. Integrating digital solutions across inpatient and outpatient settings, health care professionals can assist patients during outpatient visits, during hospitalization, and after discharge, potentially improving continuity of care [10].

Despite these opportunities, evidence on the overall impact of digital care pathways remains limited, particularly regarding resource use and costs [11]. Understanding these effects is essential from a service structure perspective, as it determines whether digital solutions truly enhance care efficiency and value.

Digital care pathways can be evaluated through the quintuple aim framework, which emphasizes population health, patient experience, care team well-being, health equity, and cost reduction [12]. Previous studies have found some benefits when digital pathways complement conventional treatment, especially for chronic conditions. For example, a Swedish study found that two-thirds of patients with chronic obstructive pulmonary disease and/or those with chronic heart failure considered a digital platform combined with structured telephone support helpful [13]. Similarly, patient satisfaction with digital services is generally high [6,14-16], and these services may promote health equity among those able to use them [17]. Health care professionals have observed that digital care pathways can help manage patient expectations and provide clinicians with predictive insights [4].

However, the impact on health care use and costs remains unclear. Some studies report increased service use following digital pathway implementation [18], while others show cost savings and improved efficiency [19,20]. It remains unclear whether digital services substitute for traditional health care encounters or add to overall service use. These small studies, which focused on individual conditions and reported mixed findings, suggest that the effects vary by condition, digital solution, and context.

To address this gap, this study focuses on digital care pathways designed to reduce or replace elective visits, emergency department visits, or phone appointments. Specifically, we examine the 10 most frequently used digital care pathways at Finland’s largest public hospital (HUS Helsinki University Hospital; hereafter, HUS) and assess how the increase of digital services is associated with contact volume, contact distribution, and outpatient costs. The following research questions were addressed: (1) How is the implementation of digital care pathways associated with the number and distribution of contacts? (2) How has the total number of contacts per patient evolved? (3) How has the contact distribution changed over time? (4) How have outpatient care costs per patient developed?

Understanding these associations is essential for health care systems seeking to optimize digital solutions for efficiency, equity, and sustainability.

## Methods

### Overview

In our retrospective longitudinal observational study, we research digital care pathways in a Finnish context, where they are defined as secure online services for patients being treated in specialized health care hospitals. The pathways form part of the Health Village portal, developed collaboratively by 5 Finnish university hospitals under the leadership of HUS within the Virtual Hospital project [21]. Digital care pathways enable patients to communicate online with health care professionals and access condition-specific information and self-care guidance. They also provide frequently asked questions, interactive exercises, questionnaires, and tools for tracking personal health data. Access requires strong authentication using online banking credentials or a mobile ID.

We analyzed the 10 most widely used digital care pathways by user volume, namely, *in vitro fertilization*, *pregnancy*, *sleep apnea*, *hip and knee osteoarthritis*, *obesity*, *inguinal hernia*, *cervical spine surgery*, *skin conditions*, *traumatic brain injury*, and *prostate cancer*. We selected only the 10 largest digital care pathways to ensure that the data pool and quality were sufficient to produce reliable results. The selection criteria were (1) more than 1500 users per digital care pathway and (2) the aim of replacing or reducing in-person contacts (Multimedia Appendix 1). Psychiatric online therapies, which represent some of the earliest forms of digital treatment solutions, were not included in our analysis. These interventions are typically classified as online therapy rather than digital care pathways. All outpatient contacts within the abovementioned diagnosis groups were included in the study.

### Data Collection

The data were obtained from the data lake of HUS. We collected the annual number of patients and contacts by contact type, covering all patients within the diagnosis group (*ICD-10* [*International Statistical Classification of Diseases, 10th Revision*]) related to the specific digital care pathway. The detailed diagnosis codes are listed in Multimedia Appendix 1. The contact types included all outpatient contacts: elective visits, emergency visits, phone calls, and digital contacts. Thus, patients were recognized as digital service users based on the registered contact type.

In addition, we collected the outpatient costs by contact type and diagnosis group annually. The costs were based on the routine cost accounting system of HUS. The unit costs included all direct and administrative costs, with the annual total costs (resources, materials, facility, administration, etc) of each department allocated to different contact types according to their use of time and other resources, following time-driven activity-based costing principles. The resulting contact-specific unit costs were used to calculate the annual costs for each diagnosis group. In the Finnish public health care system, specialized health care providers invoice the well-being services counties (formerly municipalities) based on standardized unit costs for recorded outpatient contacts. The unit costs included all direct clinical and administrative costs, with the annual departmental costs allocated to individual patient contacts.

### Data Analysis

Descriptive analyses included the total number of digital contacts, mean contacts per patient by contact type, and mean cost per patient by contact type by year. The cost per patient was calculated by dividing the total costs by the number of patients per year and per contact type. Metrics were calculated for both the pooled population and each patient group separately. Cost analyses were conducted in both nominal and real terms. Nominal costs per patient were calculated by dividing the total cost per contact type by the annual number of patients based on the HUS accounting system data. Real costs were derived by adjusting nominal values using the consumer price index published by Statistics Finland [22], with the last year of the study period set as the reference year. The consumer price index was selected to remove the effect of general inflation while preserving health care–specific cost developments that may reflect changes in productivity and service delivery.

An ecologic, group-level association analysis was conducted to assess whether the proportion of digital contacts in 2024 was related to the overall change (1) in physical contacts from 2018 to 2024 and (2) in total costs of the contacts from 2018 to 2024 across patient groups. Physical contacts included both elective and emergency visits. We used the Spearman rank correlation coefficient (ρ) to evaluate the monotonic relationship between digital contact share and the absolute change in total contacts. In addition, 95% CIs and *P* values were computed using Fisher *z*-transformation and a *t*-distribution–based test of the null hypothesis (ρ=0). The statistical tests were performed using SPSS (version 30.0.0.0; IBM Corp).

To account for the clustered longitudinal structure of the data, generalized estimating equations (GEEs) were fitted using annual pathway-level observations. The unit of analysis was a digital care pathway in a given calendar year, resulting in 70 pathway-year observations from 10 digital care pathways, followed annually between 2018 and 2024. Annual physical outpatient contacts per patient served as the dependent variable. Calendar year and the annual proportion of digital care pathway contacts were included as continuous covariates. Correlation between repeated observations within each pathway was modeled using a first-order autoregressive working correlation matrix.

STROBE statement was followed when reporting the study (Multimedia Appendix 2).

### Ethical Considerations

According to Finnish legislation, this study did not require research ethics committee approval [23], as the study involved only adult participants, the study did not entail any intervention affecting the participants’ physical or psychological integrity, and the register-based data were obtained in pseudonymized form from a data repository rather than collected directly from patients [24].

## Results

The annual number of digital care pathway users increased in total and in most patient groups (Table 1), exceeding 20,640 users in 2024. The number of digital care pathway users was greatest among patients with pregnancy, sleep apnea, hip osteoarthritis, and skin conditions, while the proportion of patients participating in digital care pathways was highest among patients with cervical spine (554/1293, 42.8%) and pregnancy (15,444/49,447, 31.2%).

Figure 1 represents the average number of visits, phone contacts, and digital care pathways per patient over the years. The total number of contacts per patient for these conditions increased by 7.6% (1.42/1.32) between 2018 and 2024. Physical visits per patient decreased by 22% (0.71/0.91), and phone visits per patient increased by 21.9% (0.39/0.32). The share of digital care pathway contacts increased from 0 to 0.3 per patient, representing 19.1% (22,018/182,188) of the total contacts among the studied patient groups. The share of physical visits decreased from 74.8% (99,201/132,622; 2018) to 54.1% (98,642/182,188; 2024).

Figure 2 illustrates a strong inverse association between the proportion of digital care pathways among total visits (x-axis, 0%-40%) and the relative change in physical visits (y-axis, ranging from 0 to –0.45). The Spearman correlation suggested a moderate negative ecologic association between digital contact proportion and total contact growth (ρ=–0.868, 95% CI –0.969 to –0.438; *P*<.001). As a complementary longitudinal analysis, GEEs were fitted to annual pathway-level observations. Calendar year was significantly associated with physical outpatient contacts (*P*<.001), indicating a gradual decline in physical contacts over the study period. After adjustment for calendar year, the annual proportion of digital pathway contacts remained significantly associated with physical outpatient contacts (*P*=.02).

**Table 1:** Annual number of digital care pathway users in the included patient groups.

Digital care pathways	Digital care pathway contacts per year (total contacts)								Total per digital care pathway
	2018	2019	2020	2021	2022	2023	2024		
Pregnancy	1512 (32,734)	10,045 (41,612)	7677 (42,048)	8464 (42,485)	9240 (39,394)	16,170 (47,621)	15,444 (49,447)	68,552 (295,341)	
Sleep apnea	46 (22,961)	354 (31,262)	1294 (35,510)	3494 (39,898)	4834 (40,727)	6427 (42,348)	5576 (40,910)	22,025 (253,616)	
Hip and knee osteoarthritis	12 (15,353)	1888 (16,079)	1974 (14,951)	2588 (18,285)	3012 (15,523)	4267 (17,196)	5441 (17,618)	19,182 (115,005)	
Skin conditions	—^a^ (32,413)	75 (32,500)	66 (29,510)	2324 (37,991)	6681 (40,598)	4297 (39,600)	3860 (39,490)	17,303 (252,102)	
Prostate cancer	— (9488)	— (7777)	— (5734)	353 (missing)^b^	2116 (12,536)	2047 (12,948)	1378 (12,808)	5894 (61,291)	
In vitro fertilization	34 (5539)	267 (5348)	502 (6031)	1073 (7056)	884 (7018)	762 (7346)	1079 (8200)	4601 (46,538)	
Traumatic brain injury	14 (3456)	273 (3240)	310 (3156)	667 (3416)	545 (3172)	736 (3000)	714 (3119)	3259 (22,559)	
Cervical spine surgery	24 (1832)	198 (2118)	168 (1365)	745 (1532)	498 (1388)	436 (1406)	554 (1293)	2623 (10,743)	
Obesity	68 (5906)	472 (6092)	464 (5392)	483 (6479)	790 (6356)	539 (6358)	412 (5867)	3228 (42,450)	
Inguinal hernia	46 (2940)	204 (3115)	130 (2491)	323 (2742)	220 (2989)	241 (3668)	379 (3436)	1543 (21,381)	
Total per year	244 (132,622)	3932 (149,143)	5409 (146,188)	13,100 (159,844)	20,759 (169,701)	21,878 (181,497)	22,018 (182,188)	87,340 (1,121,026)	

^a^Not applicable.

^b^Missing data.

**Figure 1:**
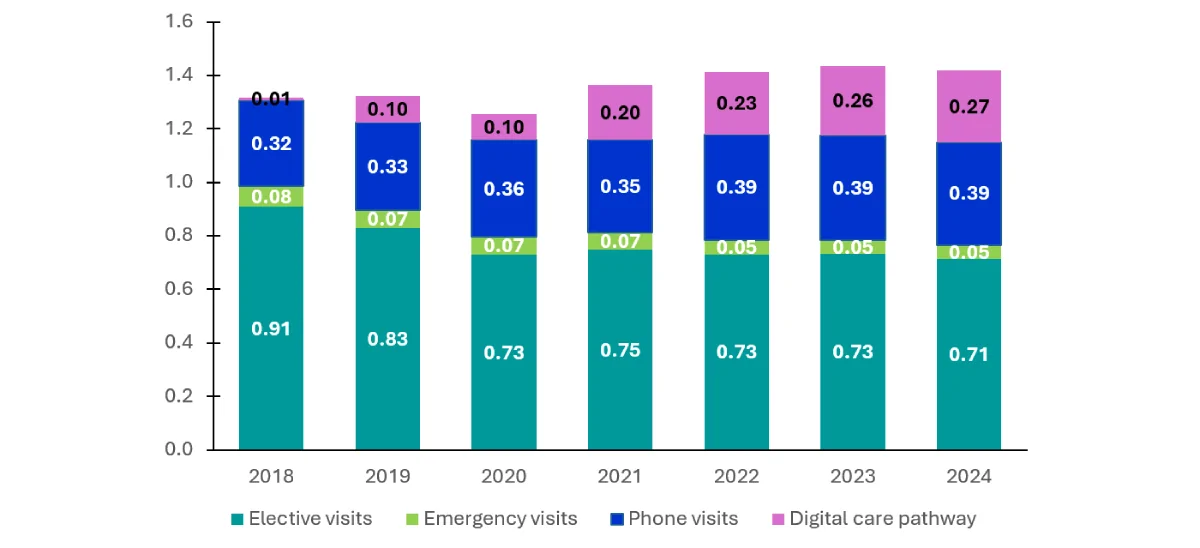
Average number of contacts per patient per contact type among the studied patient groups in digital care pathways.

**Figure 2:**
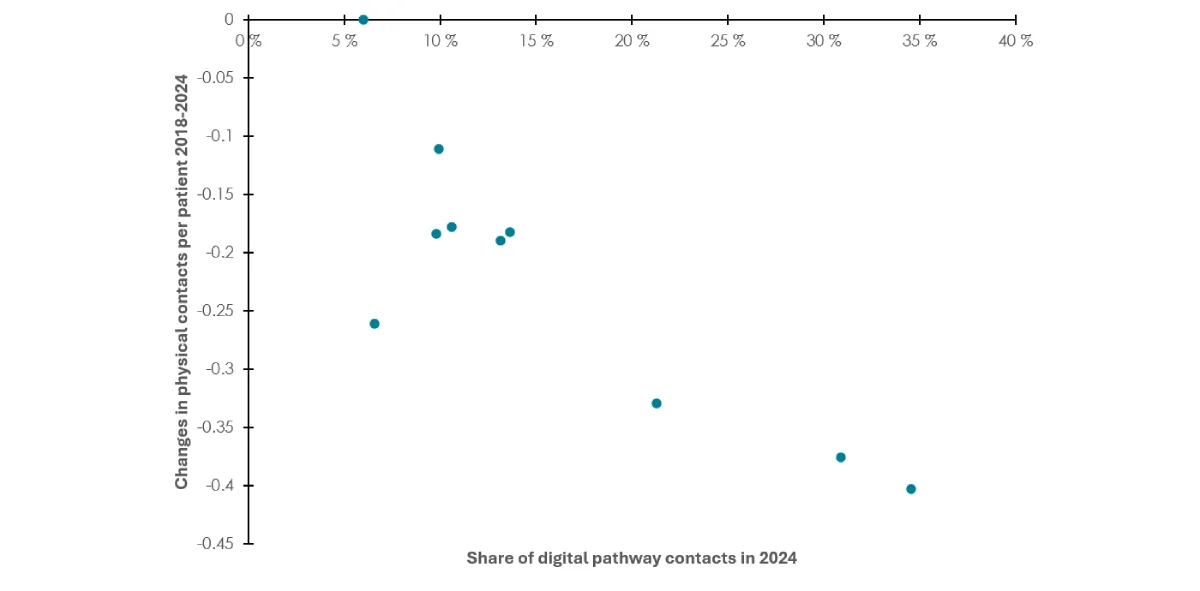
Share of digital care pathway contacts in 2024 and change in physical contacts per patient from 2018 to 2024 among the studied patient groups.

The average cost of outpatient contacts per patient decreased by 18.2% (€421/€515; €1=US $1.12 as of Oct 16, 2026) in real costs (€421/€430; 2% in nominal costs; Figure 3). The costs of physical contacts decreased by 39.3% in real costs (€233/€384; €233/€321, 27.4% in nominal costs), and the costs of phone visits increased by 15.4% (€135/€117; €135/€98, 37.8% in nominal costs). The digital care pathway contacts represented 12.6% (€53/€421 per patient) of total outpatient costs among the included patient groups in 2024.

Figure 4 shows the relationship between the proportion of digital visits among total visits (x-axis, 0%-40%) and change in real outpatient costs per patient (y-axis, ranging from −35% to 10%). The Spearman correlation suggested a nonsignificant association between digital contact proportion and changes in outpatient costs per patient (ρ=–0.239, 95% CI –0.749 to 0,470; *P*=.51). In addition, GEE analysis showed that neither calendar year (*P*=.33) nor the annual proportion of digital care pathway contacts (*P*=.22) was associated with annual outpatient costs per patient.

**Figure 3:**
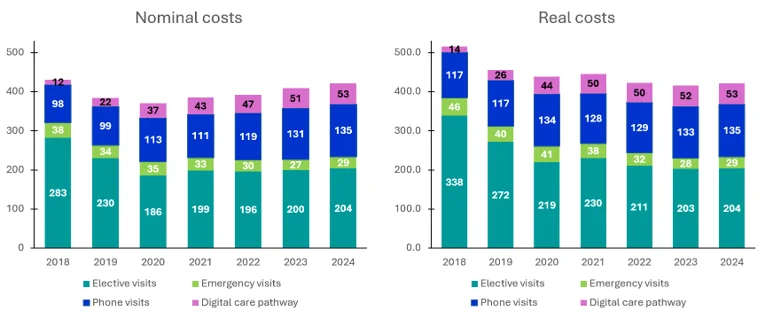
Nominal and real costs of the outpatient contacts per patient by contact type among the studied patient groups. Real costs were produced by deflating the nominal costs to the base year 2024 using the Finnish consumer price index.

**Figure 4:**
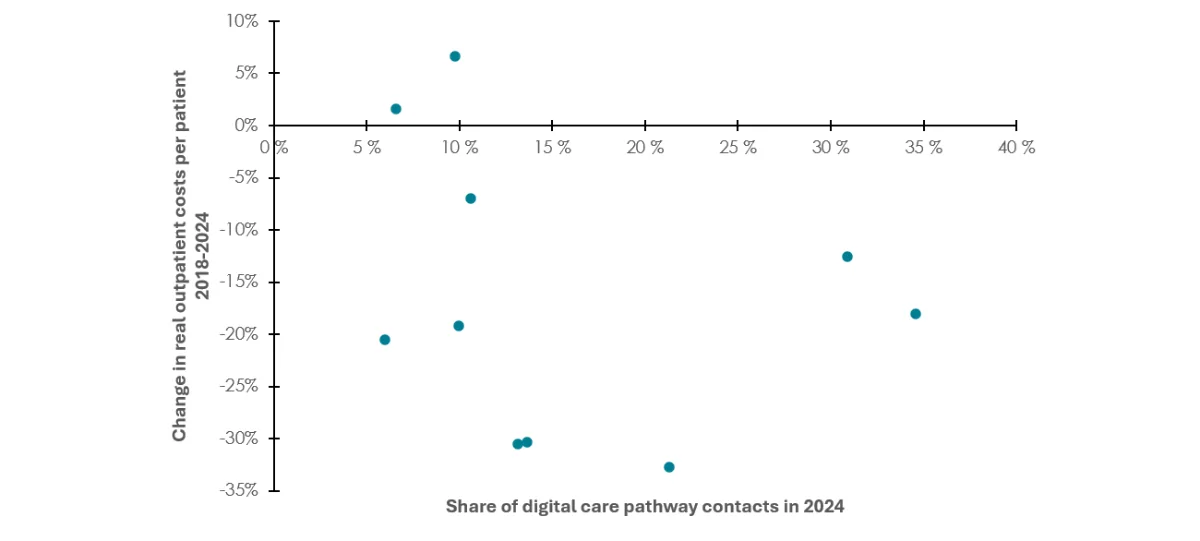
Share of digital care pathway contacts in 2024 and change in real outpatient costs per patient from 2018 to 2024 among the studied patient groups.

## Discussion

### Principal Findings

This study demonstrates a substantial increase in the use of digital care pathways in outpatient care, with the number of active users surpassing 22,000 across 10 largest patient groups using digital care pathways by 2024. This number represents 19.1% (34,837/182,188) of the total outpatient contacts in these conditions. As the share of phone contacts has also been increasing, the share of nonphysical contacts was almost half of the total contacts in 2024. These findings reflect a broader trend in digital transformation within outpatient care, where structured digital pathways offer possibilities to replace physical contacts in specialized health care. Another concurrent driver of change was COVID-19, which made it necessary to replace face-to-face visits with digital alternatives and has also been identified as a driver in other studies [25].

The use of emergency services decreased significantly in our study. Overall, digital health services appear to have considerable potential to reduce unscheduled health care visits [26-28]. However, this effect seems to vary depending on the specific technologies and patient populations involved. In our study, digital care pathways effectively reduced visits, whereas Vesinurm et al [18] observed an increase in the overall service use following the introduction of digital care pathways among patients with multiple sclerosis treated at the same hospital as in the present study. Therefore, it is crucial to identify and understand the mechanisms through which digital services, such as care pathways, exert their effects when conducting comparative research.

We observed a clear inverse association between the proportion of digital care pathway use and the number of physical outpatient visits across patient groups. While the retrospective nature of the data does not allow causal inference, the consistency and magnitude of this relationship suggest that digital care pathway implementation may play a meaningful role in decreasing reliance on face-to-face encounters. This is plausible, given that the selected digital care pathways were specifically designed to replace one or more physical visits within the care process. Thus, although this study cannot establish causality, the findings indicate that digital care pathways may contribute to tangible shifts in care delivery, as digital visits already represent almost 20% of the total outpatient contacts in the selected patient groups. Our study shows that structured digital follow-up or education can substitute routine in-person contacts. Prior research supports this interpretation, consistently showing that digital care solutions can reduce reliance on in-person visits. Large-scale analyses link telemedicine to substantial declines in outpatient encounters and e-visits, reducing new patient intake, while trials and modeling studies demonstrate that digital follow-up decreases postoperative clinic visits and no-show rates [29,30].

In contrast to the clear association with reductions in physical visits, no comparable association was observed between the proportion of digital care pathway use and total outpatient costs. Several factors may explain this. First, unit costs for digital pathways varied considerably due to differences in service models and associated cost structures. In addition, many services were newly developed, and investment-related expenses may have differed across patient groups. Second, the relatively small number of digital care pathways in the analysis (n=10) limits the analyses. Third, substantial changes in telephone visit volumes may have influenced cost structures independently of digital care pathway use. A more granular economic assessment at the pathway level is therefore needed to understand how individual digital care pathways affect cost efficiency and the mechanisms underlying these effects. Nevertheless, the observed decline in real outpatient costs per patient suggests that overall cost efficiency in outpatient care may have improved, even if the specific contribution of digital care pathways remains uncertain.

Remarkably, total outpatient contacts per patient increased slightly over the study period. This may reflect how digital care pathways and telephone consultations address only part of a patient’s requirements. While these channels appear to substitute a portion of traditional visits, a subset of patients may still require in-person assessment, generating a hybrid sequence of digital and physical encounters. This underscores the importance of continuously measuring how different contact types combine within care episodes and identifying when digital services effectively resolve care needs vs when they lead to additional steps. Such insight is essential for targeting digital care pathways appropriately and for improving their design to better meet clinical needs without unnecessary escalation to physical care.

Future studies should aim to collect data that enables a comprehensive evaluation of the cost-effectiveness of digital care pathways. Currently, cost-effectiveness is primarily indicated by reduced visits or costs, but clinical parameters, patient-reported outcome measures, and health care professionals experience should also be considered [31].

### Limitations

This study has several limitations that should be considered when interpreting the findings. First, registries rely on routine clinical documentation, which can contain missing or erroneous entries. However, diagnosis coding in Finland is highly accurate because diagnoses are always coded by the treating physician [32]. For example, the positive predictive value of the sleep apnea diagnosis is greater than 98% [33]. Second, the retrospective and ecologic nature of the data limits causal inference and result interpretation. A key limitation of this study is the absence of a concurrent nondigital comparator group, which restricts the ability to directly compare outcomes between digital and traditional intervention approaches. Third, the analysis was limited to 10 most attended digital care pathways, which restricts generalizability and may not capture the full diversity of outpatient care processes. There were dozens of smaller digital care pathways in various stages of development and implementation during the study period. However, the 10 included pathways represent around half of all patients participating in any digital care pathway at the HUS. Fourth, cost analyses were constrained by the heterogeneous allocation of development and investment expenses across pathways, as well as concurrent changes in telephone visit volumes, making it difficult to isolate the economic impact of digital pathways. In addition, the study could not account for multimorbidity or changes in case mix. Although the diagnosis definitions were designed to capture health care use related to each clinical pathway as comprehensively as possible, future patient-level studies are needed to examine how multimorbidity and overlapping care pathways influence health care use and costs. In addition, the included digital care pathways were intentionally heterogeneous, covering different clinical conditions and care models. Some pathways were primarily designed to replace scheduled outpatient visits, whereas others aimed to reduce the need for visits through patient education, self-management, or remote follow-up. Consequently, variation between pathways is expected, and the pooled analyses should be interpreted as reflecting overall implementation patterns rather than identical effects across individual digital care pathways. Finally, the study lacked patient-level clinical outcomes and patient-reported measures, which are essential for assessing the quality and effectiveness of care beyond contact volumes and costs.

### Conclusions

Digital care pathways expanded rapidly in specialized outpatient care, accounting for nearly one-fifth of all outpatient contacts in the included patient groups by 2024. Greater adoption of digital care pathways was associated with a lower proportion of physical visits, whereas no corresponding association was observed with outpatient costs.

While digital care pathways appear to have an important role in service delivery, the aggregated dataset does not allow the determination of whether digital contacts replaced or complemented specific physical contacts. Therefore, conclusions regarding substitution between digital and in-person care should be interpreted with caution. As digital care pathways become an established component of routine care, future studies should determine their effectiveness, cost-effectiveness, and impact on patient outcomes using patient-level longitudinal or controlled study designs.

## Appendix

Multimedia Appendix 1 Digital care pathways, ICD-10 (International Statistical Classification of Diseases, 10th Revision), interactivity model, and mechanisms.

Multimedia Appendix 2 STROBE checklist.

## Abbreviations

GEE: generalized estimating equation
HUS: HUS Helsinki University Hospital
ICD-10: International Statistical Classification of Diseases, 10th Revision
STROBE: Strengthening the Reporting of Observational Studies in Epidemiology
VTR: State Research Funding

## Data Availability

The datasets generated or analyzed during this study are available from the corresponding author on reasonable request.
