# Supplementary material for: Implementing Digital Care Pathways and Changes in Contact Volume, Contact Distribution, and Outpatient Costs in Specialized Health Care: Retrospective Longitudinal Observational Study
Source: J Med Internet Res. 2026 Oct 27;28:e97853. doi: 10.2196/97853 (PMC13752594; doi:10.2196/97853)
Supplement: Supplementary file 1 [file jmir_v28i1e97853_app1.docx]

Appendix 1. Digital care pathways, ICD-10, interactivity model and mechanisms.

| **Digital care pathway** | **Patient inclusion /ICD-10** | **Mechanisms** |
| --- | --- | --- |
| Sleep apnoea | G47.3 Sleep apnoea | Replaces visits |
| Hip/knee osteoarthritis | M16 Osteoarthritis of hip  M17 Osteoarthritis of knee | Replaces visits |
| Skin conditions | L00-L99 Diseases of the skin and subcutaneous tissue | Replaces visits |
| Pregnancy | Z32.1 Confirmed pregnancy | Decreases visits |
| Prostate cancer | C61 Malignant neoplasm of prostate (Prostate cancer) | Decreases visits |
| In vitro fertilisation (IVF) | N97 Female infertility  Z31.2 Encounter for in vitro fertilisation (IVF)  Z31 Encounter for procreative management (Procedures to assist reproduction)  N46 Male infertility | Decreases visits |
| Brain injury | S06 Intracranial injury | Decreases visits |
| Cervical Spine Surgery | M50: Cervical disc disorders  M54.2: Cervicalgia (neck pain) | Replaces visits |
| Obesity | E66 Obesity | Replaces visits |
| Inguinal hernia | K40 Inguinal hernia | Replaces and decreases visits |
